# Longitudinal trajectories of neurologic symptoms and cognitive associations at 36-months post-hospitalization for COVID-19

**DOI:** 10.3389/fneur.2026.1882710

**Published:** 2026-08-05

**Authors:** Eunyoung A. Lee, Maria Paula Maziero, Livia C. Merrill, Gabriela D. Colpo, Lucy Couture, Lynae Baskin, Ana E. Cahuiche, Ana Petway, Huihui Fan, Elizabeth Reese, Kendra M. Anderson, Louise D. McCullough, Paul E. Schulz, Guadalupe Javier Ortiz

**Affiliations:** 1 Department of Neurology, John P and Katherine G McGovern Medical School, The University of Texas Health Science Center at Houston, Houston, TX, United States; 2 Department of Stem Cell Transplantation Research, The University of Texas MD Anderson Cancer Center, Houston, TX, United States

**Keywords:** BrainCheck, COVID-19, long COVID, Neuro-PASC, SARS-CoV-2, neurologic symptoms, cognition

## Abstract

**Background:**

A substantial proportion of individuals hospitalized for COVID 19 experience persistent neurological and psychiatric symptoms. The long-term trajectories of these symptoms, collectively referred to as neuropsychiatric manifestation of Post-Acute Sequelae of COVID-19 (Neuro-PASC), and their association with cognitive outcomes remains poorly understood. We conducted a 36-month longitudinal cohort study to characterize Neuro-PASC symptom trajectories, examine their associations with domain specific cognitive performance, and identify baseline biological predictors of long-term neurological outcomes.

**Methods:**

We performed a longitudinal observational study of 227 adults ages 19–79 (mean = 47.6), previously hospitalized for COVID-19 symptoms. Participants completed follow-up assessments at 3/6-, 12-, 24-, and 36-months post-hospitalization. At each visit, structured, self-reported neurological symptom surveys, digital cognitive testing (BrainCheck), and updated medical history were collected. Generalized estimating equation models assessed longitudinal associations between cognitive subdomains and individual symptoms. K-means clustering identified distinct symptom trajectory groups. Baseline demographic and clinical factors were examined and compared across symptom trajectory groups.

**Results:**

Several neurological symptoms increased in prevalence over 36 months following hospitalization in some individuals. Fatigue remained most common (53–65%), followed by memory/concentration difficulties (47–61%) and sleep disturbances (40–48%). Dizziness had a modest increase (30–49%), while coordination impairment more than doubled (18–41%). Headaches remained prevalent and relatively stable (21–35%). Symptom co-occurrence strengthened over time, with fatigue frequently clustering with other symptoms (up to 45% at 36 months). Overall BrainCheck composite scores were not independently associated with neurological symptoms, but significant associations emerged for executive function, memory, and attention, including time-dependent effects. Trajectory analyses identified distinct longitudinal patterns (1. stable trajectory, 2. worsening trajectory, and 3. improving trajectory), associated with baseline demographics, comorbidities, and acute disease severity.

**Conclusion:**

Neurological symptoms can persist or worsen up to 36 months post- COVID-19 hospitalization, even as some cognitive functions show partial recover. Distinct symptom trajectories and evolving associations with specific cognitive domains and biological outcomes underscore the heterogeneous and dynamic nature of Neuro-PASC, supporting the need for long-term neuropsychological monitoring and informing targeted interventions and mechanistic studies.

## Introduction

Severe Acute Respiratory Syndrome Coronavirus 2 (SARS-CoV-2), the virus responsible for COVID-19, has caused an unprecedented global health crisis, resulting in nearly 780 million confirmed infections and over 7 million deaths worldwide (1). While initial efforts focused on mitigating acute respiratory complications (2–4), accumulating evidence highlights the long-term consequences of COVID-19, collectively termed Post-Acute Sequelae of COVID-19 (PASC), or long COVID (5–7). Affecting approximately 10–20% of survivors (8, 9), PASC encompasses a range of persistent symptoms that significantly impair quality of life and pose an emerging public health challenge (10–12). Importantly, PASC is heterogeneous and evolves over time, with variable onset, persistence, and recovery across individuals (13, 14), underscoring the need for longitudinal, trajectory-based evaluation rather than cross-sectional descriptions.

Among these sequelae, neurological and psychiatric manifestations, commonly referred to as Neuro-PASC, are among the most prevalent and disabling. They substantially impair daily functioning and, in some cases, persist or worsen for years after initial infection (15–19). Frequently reported neurological symptoms include fatigue, memory/concentration difficulties, impaired coordination, headaches, dizziness, and sleep disturbances (13, 17, 20). Some reports propose that these dynamic symptom patterns suggest underlying biological heterogeneity, potentially involving different mechanisms, or their interaction (21, 22); however, definitive mechanisms remain to be elucidated. These symptoms are most prevalent among COVID-19 survivors with chronic comorbidities, specific demographic risk factors, greater acute illness severity, and hospitalization due to COVID-19 (23–25).

Hospitalized COVID-19 survivors represent a clinically and biologically high-risk subgroup with greater inflammatory burden and exposure to critical care stressors (26, 27), warranting focused longitudinal study, while acknowledging potential limits to generalizability. Of note, the presence of these neurological symptoms has been associated with an increased risk of anxiety and depression (13, 28). However, it remains unclear why only a subset of individuals develops persistent neurological manifestations or the mechanisms through which these symptoms may eventually resolve or worsen.

Cognitive deficits are a common manifestation of Neuro-PASC and may persist for months or even years following SARS-CoV-2 infection (13, 29, 30). Similar to other neurological symptoms, the increased prevalence of cognitive impairment has been associated with pre-existing comorbidities, greater severity of acute COVID-19 illness, and hospitalization (30–32). Reported deficits frequently involve attention, memory, executive function, and processing speed, domains that are critical for daily functioning and quality of life (33–35). Despite their frequent co-occurrence (36–38), most prior studies have assessed neurological symptoms and cognition separately or cross-sectionally (39–41), leaving the longitudinal relationships between symptom trajectories and domain-specific cognitive outcomes poorly characterized. Moreover, few investigations extend beyond 12–18 months (42–45), limiting insight into long-term persistence, progression, or recovery; thus, longer follow-up is needed to delineate durable Neuro-PASC trajectories and their cognitive correlates.

To address these gaps, we conducted a 36-month longitudinal observational study of 227 adults previously hospitalized for COVID-19. Participants completed follow-up assessments at 3/6, 12-, 24-, and 36-months post-hospitalization, including standardized, self-reported, neurological symptom surveys and cognitive testing using the *BrainCheck* digital platform. Using generalized estimating equation (GEE) models, we evaluated associations between cognitive subdomains and individual symptoms over time and applied K-means clustering to identify distinct symptom trajectory patterns. In addition, we compared baseline demographic, clinical, and available biological characteristics across trajectory groups to explore predictors of symptom course.

We hypothesized that (i) Neuro-PASC symptoms would exhibit distinct, heterogeneous trajectories over 36 months; (ii) domain-specific cognitive performance would show differential, time-dependent associations with these trajectories; and (iii) baseline clinical and biological factors would distinguish trajectory groups. This study provides novel insights into the persistence and evolution of Neuro-PASC, reveals symptom clustering patterns, and explores associative links between cognitive domains and symptom trajectories. Collectively, these findings inform long-term neurological and cognitive monitoring strategies, rehabilitation planning, and the design of mechanistic studies of post-COVID neurological dysfunction.

## Methods

### Aim, design and study population

This prospective longitudinal observational study aimed to characterize the 36-month trajectories of self-reported neurological symptoms and evaluate their associations with cognition in adults hospitalized for COVID-19. The study cohort included 630 patients admitted to Memorial Hermann Hospital at UTHealth Houston (Texas, USA) between May 2020 and August 2022 for severe COVID-19. Of these, 111 patients died during hospitalization, 28 were transferred to a long-term care or nursing facility, and 491 were discharged home. SARS-CoV-2 infection was confirmed in all patients by real-time polymerase chain reaction (RT-PCR). Eligible participants were adults 18 years or older at the time of enrollment. Medical records were reviewed, and individuals with documented histories of cognitive decline or psychiatric disorders prior to hospitalization were excluded. Written informed consent was obtained from all participants or their legally authorized representatives.

### Data collection

Following hospital discharge, 227 patients returned for follow-up evaluation and completed at least one valid longitudinal assessment and were included in follow-up analyses. Study baseline was defined as the time of hospital discharge, at which demographic and clinical variables were collected. Follow-up evaluations were scheduled at 3, 6-, 12-, 24-, and 36-months post-discharge; assessments conducted at 3/6 months were grouped as an early follow-up timepoint, with 12-, 24-, and 36-month assessments treated as subsequent follow-up visits. At each follow-up visit, participants completed structured, self-reported assessments of neuropsychiatric symptoms, underwent an updated medical history review, and participated in neurocognitive testing. Participant retention was monitored throughout the study period. Among participants with at least one follow-up assessment, 83 completed one visit, 80 completed two visits, 41 completed three visits, and 23 completed four visits. All analyses were conducted using available data from follow-up time points.

### Self-reported neuropsychiatric symptoms

As in previous COVID-19 studies (46–48), study participants completed a brief structured interview at each follow-up visit to assess self-reported neuropsychiatric symptoms. Participants responded yes/no to the presence of impaired coordination, dizziness on standing, fatigue or reduced ability to perform usual activities, headache, memory/concentration difficulties, and sleep disturbances.

Depressive symptoms were assessed using the Patient Health Questionnaire-9 (PHQ-9; score range 0–27) and anxiety was assessed using the Generalized Anxiety Disorder-7 (GAD-7; score range 0–21). Both instruments assess symptom frequency over the preceding 2 weeks using a 4-point response scale (0 = not at all to 3 = nearly every day) and were administered at each follow-up visit from 3 to 6 through 36 months. The PHQ-9 and GAD-7 demonstrate strong internal consistency and sensitivity to change over time (49, 50). In analyses, PHQ-9 and GAD-7 total scores were treated as continuous variables to capture symptom severity.

### Cognitive assessments

Digital cognitive tools provide a sensitive and scalable method for objectively identifying subtle cognitive changes, often earlier than conventional assessment approaches (51). Cognitive function was evaluated using BrainCheck, a validated, self-administered FDA Class II digital cognitive battery that evaluates multiple cognitive domains within 10–15 min (52). Its secure remote-testing capability enabled participants to complete assessments without in-person visits, an important advantage during the COVID-19 pandemic.

*The BrainCheck* battery has several standardized tasks, including the Immediate and Delayed Recognition Tests (IRT/DRT) for short- and long-term memory, the Stroop Color and Word Test (SCWT) for cognitive flexibility, the Digit-Symbol Substitution Test (DSST) for processing speed, and Trail Making Tests A and B (TMTA/TMTB) for attention and executive function (52).

*BrainCheck* has demonstrated moderate to strong performance correlations (53), high sensitivity in cross-sectional and test–retest studies (54, 55), and the ability to distinguish between normal cognition, mild cognitive impairment, and dementia (56). *BrainCheck* has shown greater sensitivity for identifying cognitive deficits in longitudinal studies relative to other digital assessments, including the NIH Toolbox, and has outperforms traditional assessments such as the Dementia Severity Rating Scale (DSRS) in detecting cognitive changes (57, 58).

The overall *BrainCheck* composite score is derived by averaging all subtest scores except TMTB, which is excluded to prevent missing data resulting from incomplete TMTA performance (54). Raw subtest scores were standardized for age and device type, producing scaled scores with a mean of 100 and a standard deviation of 15. For this analysis, we used the corresponding normed scores ranging from 0 to 200. Consistent with validated thresholds (56, 58), scores below 85 were classified as suggestive of possible cognitive impairment, whereas scores of 85 or higher indicated that impairment was unlikely. BrainCheck employs an internal algorithm to flag abnormal performance using predefined criteria. All performance validity tests, except one, met the validity criteria.

### Statistical analysis

Demographic characteristics, depression and anxiety scores, and cognition testing throughout follow-up were summarized using *n* (%) for categorical variables and median [interquartile range, IQR] for continuous variables. The prevalence of each neurological symptom and cognitive score was assessed at each follow-up visit to examine their changes over time.

To investigate the association between cognition and neurological symptoms longitudinally, a generalized estimating equation (GEE) model was applied. Two models with varying covariates were developed: (1) cognition, time, and their interaction; (2) cognition, depression, anxiety, time, age, sex, and interactions of time with cognition, depression, and anxiety. Additional interaction terms were tested to control for Hispanic ethnicity, obesity, diabetes, and hyperlipidemia. Analyses were performed for both overall cognition and individual cognitive subdomains. The *BrainCheck* overall score was used as a measure of cognitive function. Additionally, its subscale scores (e.g., attention, executive function, and memory) were analyzed separately to explore the effects of different cognitive domains on the associations identified.

To determine whether neurological symptoms were correlated or co-occurred, co-occurrence rates and tetrachoric correlations were computed at each follow-up. Co-occurrence rates measured the absolute frequency of two symptoms appearing together and indicated how often both symptoms were present simultaneously. Correlations assessed the strength of the relationship between two symptoms. *p*-values for correlation were adjusted by FDR.

K-means clustering was conducted for each symptom to identify groups of subjects with similar trajectories over time, where the dimensions were the follow-up time points. Clustering results were evaluated using validation metrics such as within-cluster sum of squares, between-cluster sum of squares, Silhouette score, and the Calinski-Harabasz index. Based on these metrics, the optimal number (*k* = 2, 3, 4) of clustering groups was selected. To examine whether baseline demographic and clinical characteristics differed across symptom trajectory groups, chi-square tests were used for categorical variables and one-way analysis of variance (ANOVA) was used for continuous variables. Baseline characteristics included age, sex, race, Hispanic ethnicity, obesity, diabetes type II, hyperlipidemia, hypertension, asthma, acute respiratory distress syndrome (ARDS), and COVID-19 severity.

Post-hoc power analysis was conducted to evaluate the statistical power of the study. With 227 participants and an average of 2.5 observations per participant across the 36-month follow-up period, the study had approximately 80% power to detect an odds ratio of 1.5 or greater for the association between cognitive measures and neurological symptoms in the GEE models, assuming a two-sided alpha of 0.05 and a symptom prevalence of 50%. For the K-means clustering analyses, with sample sizes ranging from 19 to 172 across trajectory groups, the study had adequate power to detect medium effect sizes (Cohen’s d ≥ 0.5) for continuous variables and moderate differences (Cramer’s *V* ≥ 0.25) for categorical variables when comparing baseline characteristics across trajectory groups. All statistical analyses were performed using R statistical software (version 4.3.1; R Foundation for Statistical Computing, Vienna, Austria). A two-tailed *p* < 0.05 was considered statistically significant.

## Results

### Patient characteristics

A total of 227 post-COVID-19 patients who had at least one follow-up visit post-hospitalization were enrolled for assessment. The median age was 48 years [IQR: 38–57], and 119 patients (52%) were male. The majority of the cohort (60%) identified as Hispanic. COVID-19 severity at hospitalization was classified as moderate, severe, or critical. Severity was defined using established clinical criteria, including SpO₂ < 94% on room air at sea level, PaO₂/FiO₂ < 300 mmHg, respiratory rate >30 breaths/min, or lung infiltrates >50%. Most patients experienced moderate COVID-19 severity during their acute illness (55%). Common comorbidities included obesity (61%), diabetes (30%), and hyperlipidemia (11%). During follow-up, patients exhibited minimal anxiety, with a median GAD-7 score ranging between 3 and 4, and mild depression, with a median PHQ-9 score ranging between 5 and 5.5. Detailed data are in Supplementary Table 1.

### Prevalence of neurological symptom 36 months after COVID-19 hospitalization

**Figure 1:**
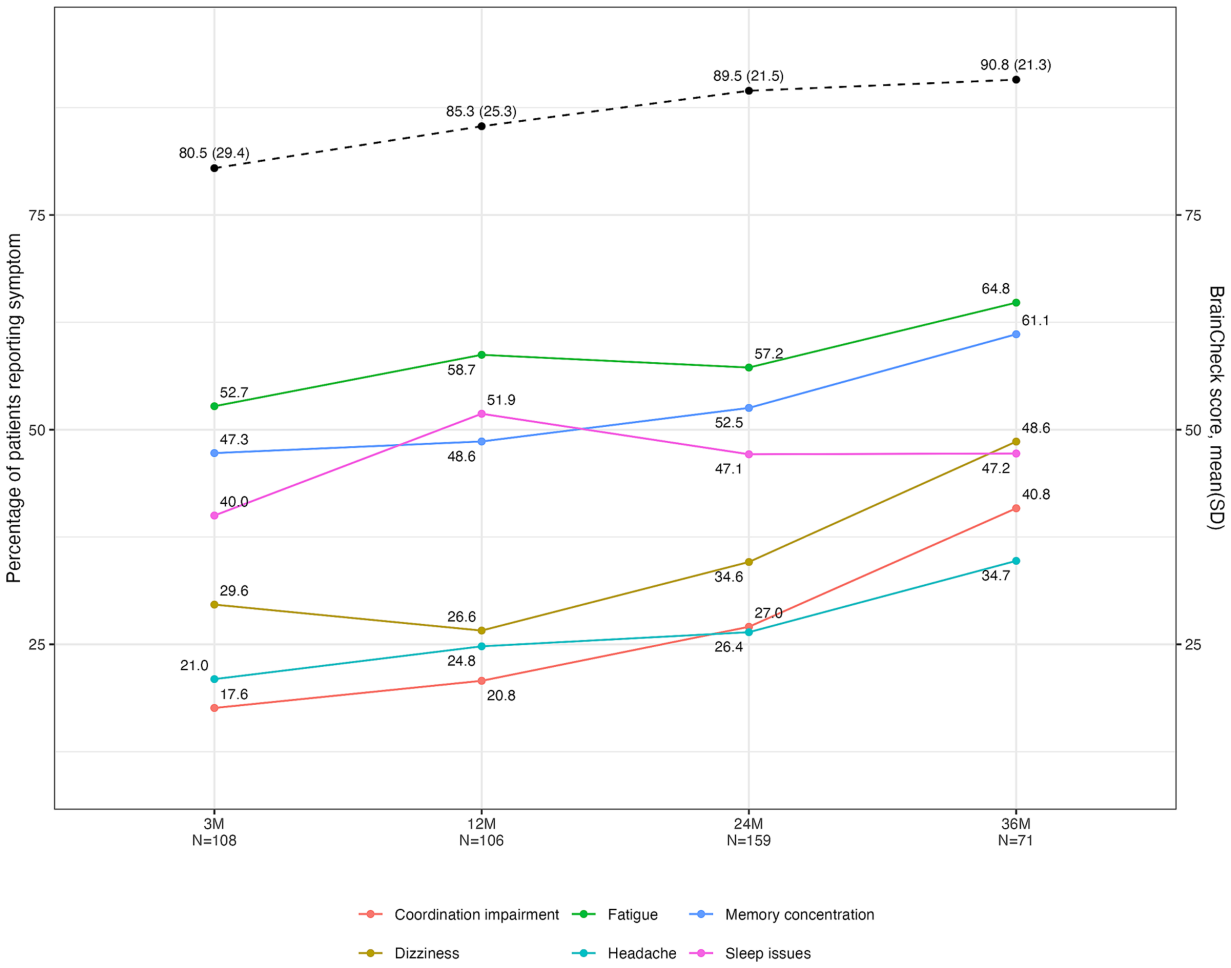
Longitudinal changes in Post-COVID self-reported symptoms, BrainCheck, anxiety and depression scores over 36 months. The figure highlights the persistence of key post-COVID symptoms despite modest improvements in objective cognitive performance, underscoring the sustained symptom burden in this cohort.

The prevalence of all neurological symptoms increased over 36 months across the cohort (Figure 1). Fatigue remained the most prevalent symptom, affecting 53% of participants at 3/6 months and increasing to 65% over 36-months. Dizziness rose substantially from 30 to 49%. Coordination impairment and headache were initially less common (18 and 21% respectively), but increased steadily, with headache prevalence reaching 35% and coordination impairment more than doubling to 41% by 36 months. Memory/concentration difficulties also increased, rising from 47 to 61%, while sleep issues fluctuated but remained relatively stable (40–48%).

### Association between cognitive performance and neurological symptoms at 36 months post-COVID-19 hospitalization

**Figure 2:**
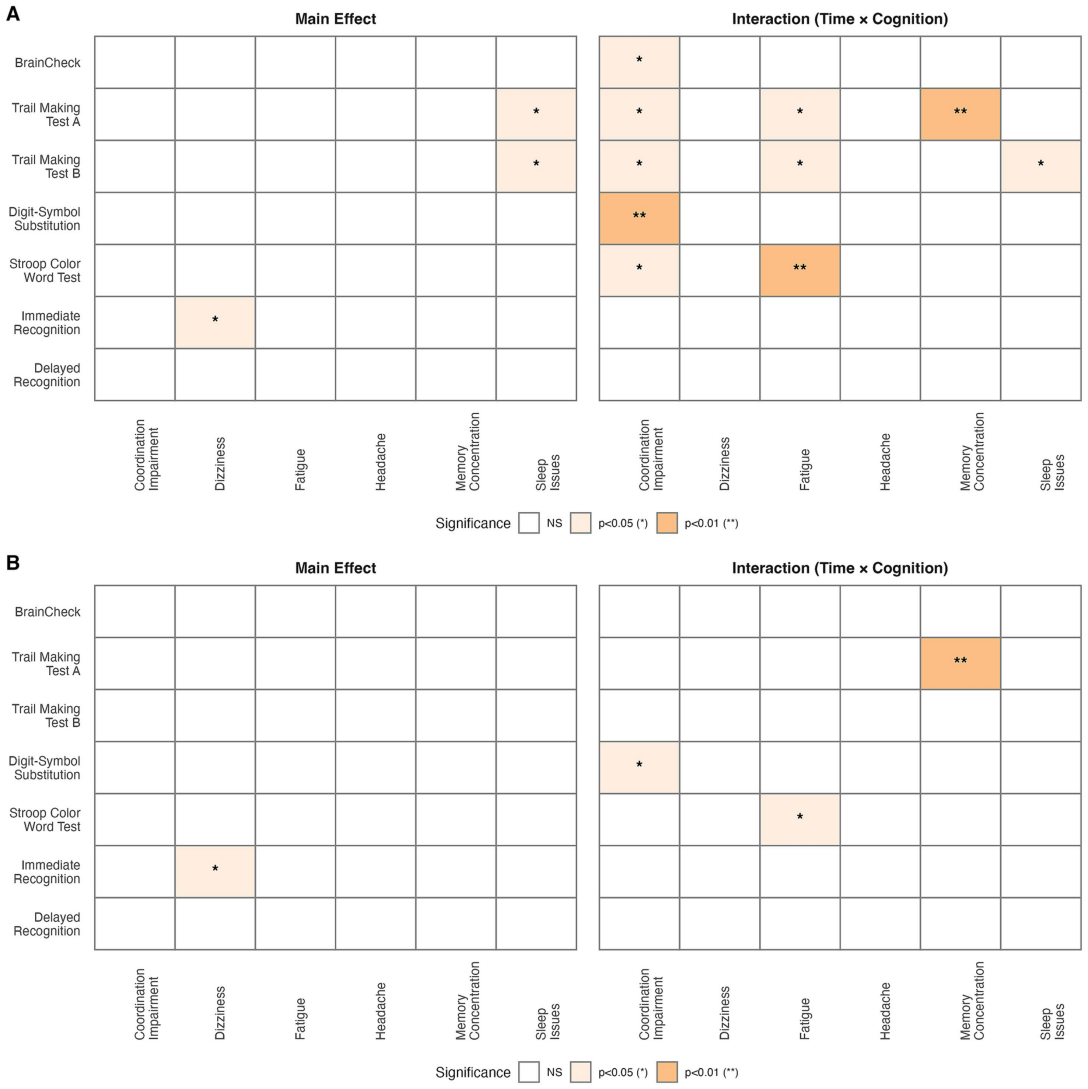
Longitudinal associations of cognitive performance test with self-reported neurological symptoms from generalized estimating equations models. **(A)** Model 1: Unadjusted. **(B)** Model 2: Adjusted for age, sex, depression (PHQ-9), and anxiety (GAD-7). All models included symptoms as the outcome, cognitive score and time as predictors, with interaction terms for time × cognition, time × PHQ-9, and time × GAD-7.

Overall *BrainCheck* composite cognitive scores showed modest improvement over time (Figure 1), suggesting partial recovery of cognitive function. However, despite these improvements, a significant proportion of patients continued to experience Neuro-PASC up to 36 months post-hospitalization. After adjustments for age, sex, depression, and anxiety, the overall *BrainCheck* score was not significantly associated with individual neurological symptoms (Figure 2, Supplementary Table 2).

In contrast, symptom prevalence increased over time for several domains, including coordination impairment (*p* = 0.014) and memory/concentration difficulties (*p* = 0.049). Higher depression (PHQ-9) burden was associated with increased odds of most symptoms (ORs = 1.17–1.23, all *p* < 0.05), except for headaches and sleep issues. Additionally, symptoms of coordination impairment (OR = 1.87, 95% CI [1.03–3.39], *p* = 0.040), headaches (OR = 1.87, 95% CI [1.08–3.25], *p* = 0.027), and memory/concentration difficulties (OR = 2.17, 95% CI [1.26–3.73], *p* = 0.005) were more likely to occur in females. Age also influenced symptom presentation, with older patients more prone to coordination impairment and fatigue, while younger patients were more likely to experience headaches (OR = 0.97, 95% CI [0.95–0.99], *p* = 0.017). Interactions with comorbidities, including obesity, diabetes, hyperlipidemia, and Hispanic ethnicity, were found to be insignificant.

Although the overall *BrainCheck* score did not reveal significant associations with non-cognitive symptom, a deeper analysis of specific cognitive domains highlighted several meaningful relationships. Coordination impairment was associated with executive function (*p* = 0.026), as measured by DSST. Dizziness was associated with memory performance assessed via the IRT (*p* = 0.015). Fatigue showed a consistent relationship with executive function measured by SCWT (*p* = 0.024), suggesting that patients experiencing fatigue may have more difficulty with cognitive tasks requiring sustained attention and response inhibition. Memory/concentration issues were associated with attention on the TRTA (*p* = 0.010). Notably, many cognitive measures showed significant interactions with time. This highlights the dynamic nature of the relationship between cognitive function and neurological symptoms during the recovery period, emphasizing that these associations are not static but change as recovery progresses.

### Co-occurrence and correlation of neurological symptoms at 36 months post-COVID-19 hospitalization

**Figure 3:**
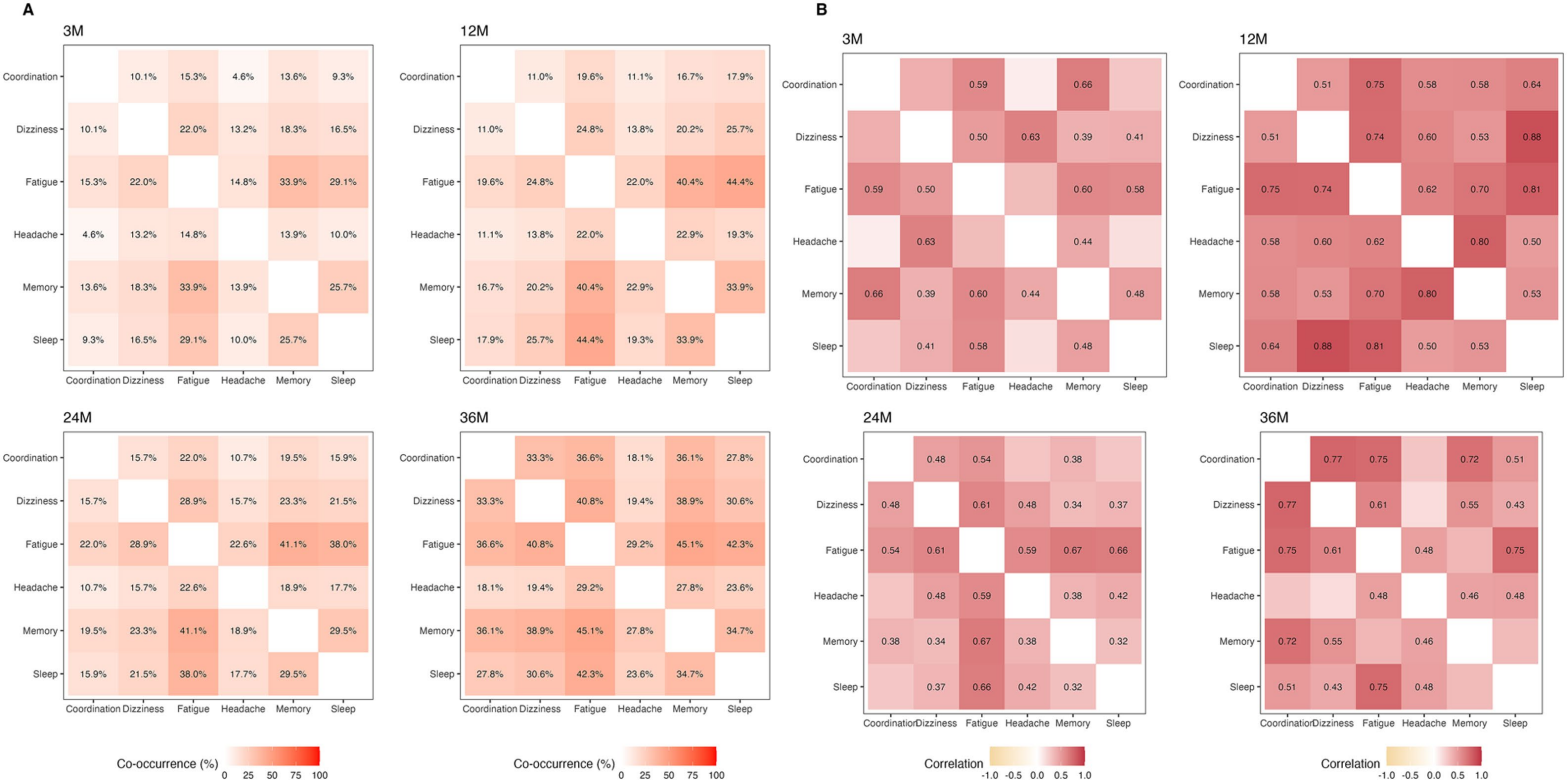
**(A)** Co-occurrence rates and **(B)** Tetrachoric correlation between self-reported neurological symptoms over 36 months. Neurological symptoms cluster and relate to each other over 36 months, with fatigue consistently co-occurring with multiple symptoms. Correlations strengthened for most symptom pairs over time, though some relationships shifted, reflecting evolving patterns of long-term post-COVID symptomatology.

Figure 3A illustrates symptom co-occurrence frequencies, and Figure 3B depicts symptom correlations over 36 months. Fatigue most frequently co-occurred with other neurological symptoms across follow-up (Figure 3A). By 36 months post-COVID-19 hospitalization, most symptoms demonstrated moderate to strong correlations (Figure 3B). During early recovery (3/6 months), headache and dizziness were strongly correlated (*r* = 0.63) despite a low co-occurrence rate (14%), reflecting their lower prevalence at this time point. The correlation between sleep disturbances and dizziness peaked at 12 months (*r* = 0.88), representing the strongest correlation observed in the study, before declining at 24 months (*r* = 0.37) and stabilizing by 36 months (*r* = 0.43). By the end of follow-up, the strongest correlation was observed between coordination impairment and dizziness.

The co-occurrence of memory/concentration difficulties and fatigue increased over time, rising from 34% at early follow-up to 45% at 36 months. Although their correlation was moderate during the first 24 months (*r* = 0.60–0.70), it declined by 36 months and was no longer statistically significant, indicating that these symptoms became increasingly prevalent but less tightly linked over time. Sleep disturbances and fatigue consistently represented the second most common symptom pair, with sustained moderate to strong correlations throughout follow-up (*r* = 0.58–0.81).

### Identification of distinct neurological symptom trajectories

**Figure 4:**
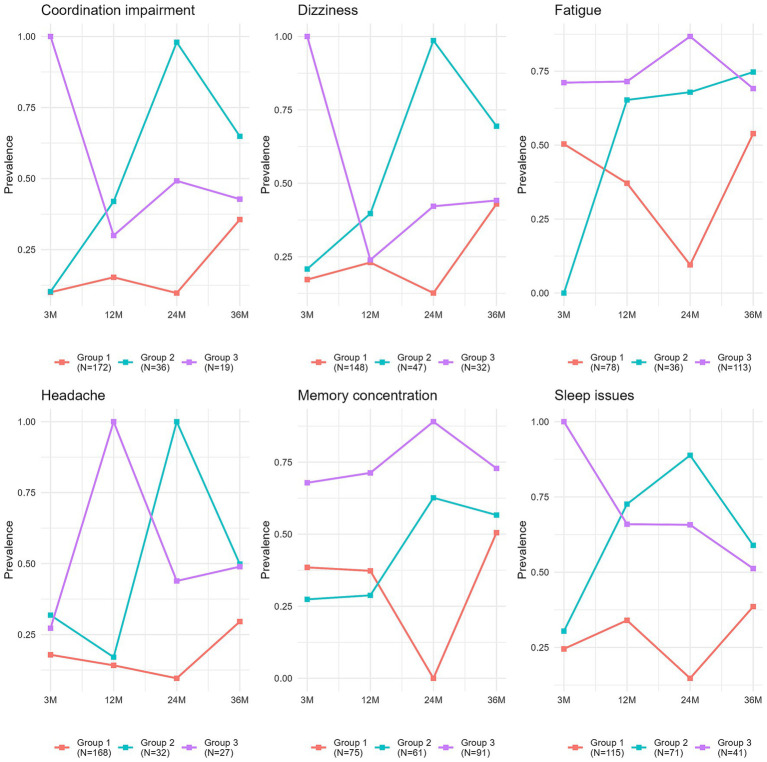
Distinct trajectory patterns of self-reported neurological symptoms identified through K-means clustering over 36 months. Three distinct groups were identified for each symptom. Clusters could represent meaningful patterns. Group 1: Stable trajectory; Group 2: Worsening trajectory; Group 3: Improving trajectory; Note the interpretation of clusters for fatigue, memory and sleep due to their lower silhouette scores.

K-means clustering was used to identify distinct longitudinal patterns of symptom evolution, yielding three trajectory groups for each symptom (Figure 4). Three patterns were identified: Group 1 are subjects who exhibited consistently low symptom prevalence; Group 2 demonstrated a worsening pattern, with symptoms increasing to a peak followed by partial recovery; and Group 3 showed high initial prevalence with subsequent improvement and stabilization that was still consistent with distress. Cluster validity metrics used to determine the optimal number of groups are provided in Supplementary Table 3. Silhouette scores for fatigue, memory/concentration difficulties, and sleep disturbances were low (≤0.3), indicating substantial overlap between clusters; therefore, these symptoms were excluded from subsequent trajectory analyses.

### Baseline characteristics associated with neurological symptom trajectories

Baseline demographic and clinical characteristics collected during hospitalization were compared across neurological symptom trajectory groups (Table 1; full results in Supplementary Table 4). For coordination impairment, participants in the consistently low trajectory (Group 1) were younger at baseline (mean age 46 years; *p* = 0.011). In contract, coordination impairment in Groups 2 and 3 was associated with higher prevalence of comorbid conditions, including type 2 diabetes (*p* = 0.024), hypertension (*p* = 0.002), and asthma (*p* = 0.006). For dizziness, the improving trajectory (Group 3) was characterized by older age (mean 53 years), higher proportion of females (69%), and greater prevalence of hypertension (56%) compared to the other groups (Age, *p* = 0.010; Sex, *p* = 0.019; and hypertension, *p* = 0.015). Disease severity during hospitalization also differed significantly across groups (*p* = 0.028). For headache, younger age was associated with the improving trajectory (Group 3; mean age 43 years) relative to Groups 1 and 2 (*p* = 0.031).

**Table 1:** Baseline characteristics associated with neurological symptom trajectories.

Characteristics	Consistently low (group 1)	Worsening (group 2)	Improving (group 3)	*p*-value
Coordination impairment				
*N*	172	36	19	
Age, mean (SD)	46.4 (12.6)	51.3 (12.9)	52.3 (14.8)	0.011
Diabetes II, *n* (%)	45 (26.2%)	18 (50%)	6 (31.6%)	0.024
Hypertension, *n* (%)	53 (30.8%)	22 (61.1%)	11 (57.9%)	0.002
Asthma, *n* (%)	11 (6.8%)	6 (17.6%)	6 (33.3%)	0.006
Dizziness				
*N*	148	47	32	
Age, mean (SD)	46.3 (13.1)	48.5 (13.3)	52.7 (11)	0.010
Male, *n* (%)	86 (58.1%)	23 (48.9%)	10 (31.2%)	0.019
Hypertension, *n* (%)	46 (31.1%)	22 (46.8%)	18 (56.2%)	0.015
Severity, *n* (%)				0.028
Moderate	68 (48.6%)	32 (72.7%)	18 (60%)	
Severe	65 (46.4%)	11 (25%)	9 (30%)	
Critical	7 (5%)	1 (2.3%)	3 (10%)	
Headache				
*N*	168	32	27	
Age, mean (SD)	48.7 (13)	45.7 (13.9)	43.4 (10.8)	0.031

Only variables with statistically significant differences (*p* < 0.05) are shown; full results including non-significant variables are presented in Supplementary Table 4.

## Discussion

This 36-month longitudinal study post hospitalization for severe COVID-19 infection provides novel insights into the persistence and evolution of neurological symptoms among individuals hospitalized for COVID-19 and advances our understanding of Neuro-PASC by addressing key limitations in prior research. Earlier studies documented persistent neurological symptoms following COVID-19 (18, 20, 59), but most were restricted to short-term follow-up and lacked integration of objective cognitive measures (40, 43, 60). Our principal findings are that (i) multiple Neuro-PASC symptoms persist and often worsen through 36 months, (ii) global cognition shows modest improvement while domain-specific associations with symptoms remain, and (iii) heterogeneous symptom trajectories can be empirically identified.

Our findings extend this literature by demonstrating that Neuro-PASC symptoms not only persist, but often worsen over time, with fatigue, memory complaints, and dizziness showing marked increases in prevalence at 36 months post-hospitalization. These patterns are consistent with chronic post-infectious sequelae described after other viral illnesses (61–63), while differing in scale and heterogeneity (61), and they underscore the chronic nature of post-COVID neurological sequelae and their potential to impact quality of life years after the acute illness.

### Factors associated with neurological trajectories

Previous investigations have reported symptom stabilization or partial improvement within the first-year post-infection (64–66). In contrast, our data reveal progressive worsening for a substantial subset of patients, highlighting the need for extended monitoring beyond 12 months. Possible explanations include delayed or relapsing inflammatory processes (67), cumulative functional burden and deconditioning (68, 69), interaction with aging or comorbidities (70), and greater symptom awareness over time; however, mechanistic inference is premature and requires dedicated study.

Furthermore, while global cognitive scores exhibited slight improvement, domain-specific associations revealed complex relationships between cognitive performance and symptom trajectories. This pattern may reflect the localized nature of brain pathology due to COVID-19. Damage to specific neural networks can produce distinct cognitive deficits (71–73), thereby shaping how self-reported symptom burden relate to particular cognitive systems. This highlights the importance of domain-level assessment rather than reliance on composite scores alone.

The identification of three distinct symptom trajectory clusters- (1) low-stable, (2) worsening with partial recovery, and (3) high-to-improving- offers a clinically meaningful framework for understanding heterogeneity in long-term outcomes. These patterns may reflect differences in underlying mechanisms, comorbidities, or resilience factors, and warrant significant additional investigation considering the distress associated with them.

### Demographic and clinical factors influencing trajectories

Analysis of baseline demographics and clinical characteristics across symptom trajectory groups revealed several noteworthy patterns. For coordination impairment, younger patients with fewer comorbidities were more likely to remain in the consistently low-symptom trajectory, whereas patients with metabolic and cardiovascular comorbidities including diabetes, hypertension, and asthma were overrepresented in worsening and improving trajectories. Consistent with previous reports (74, 75), these findings suggest that pre-existing comorbidity burden may increase vulnerability to the development and persistence of coordination impairment, and potentially other Neuro-PASC symptoms, among patients hospitalized for COVID-19.

For headache, younger age was associated with an improving symptom trajectory. This observation aligns with prior reports indicating that COVID-19-related headache is more prevalent in younger individuals (76). Together, these results indicate that age and comorbidity profiles differentially influence symptom evolution across neurological domains, reinforcing the need for risk stratification that accounts for baseline clinical context.

### Study limitations and future directions

Several limitations should be acknowledged. First, reliance on self-reported symptom data introduces potential recall bias and variability in individual patient perception, which may affect the precision of symptom burden estimates. Importantly, however, participants were assessed repeatedly over time, yielding multiple observations per subject. This repeated-measures design helps mitigate some limitations of single time-point self-report by capturing within-person variability, reducing the influence of transient states, and allowing for more reliable estimation of symptom trajectories. Future research, however, might incorporate objective clinical assessments and standardized neuropsychological evaluations alongside self-reports, as well as digital health tools (e.g., wearables for fatigue and sleep monitoring) to validate symptom burden (77).

Second, our sample consisted exclusively of previously hospitalized individuals, which may limit generalizability to those with milder acute illness. Expanding future cohorts to include community-based and non-hospitalized patients, and stratifying analyses by severity of initial infection, will provide a more comprehensive understanding of Neuro-PASC across the full spectrum of COVID-19. Relatedly, survivorship and selection effects, due to mortality, severe disability, or inability to participate, may bias estimates toward healthier long-term survivors and could lead to underestimation of symptom persistence or severity.

Third, attrition over the 36-month follow-up period may introduce selection bias as participants who remained in the study could differ systematically from those lost to follow-up. To mitigate this, future studies should implement retention strategies such as regular engagement, flexible scheduling, and remote assessments, and apply statistical techniques like inverse probability weighting to adjust for attrition bias.

Fourth, although this study does not directly investigate biological mechanisms, the observed associations between cognitive domains and symptom trajectories do suggest testable hypotheses regarding dysfunction within interconnected neural networks in Neuro-PASC. These findings suggest that distinct brain regions and circuits may underlie heterogeneous symptom evolution. Future studies incorporating multimodal neuroimaging, including structural MRI, diffusion tensor imaging (DTI), and functional imaging, along with biomarker approaches, will be essential to elucidate neural correlates and mechanistic pathways of Neuro-PASC. Such efforts will be critical for informing the development of targeted therapeutic and rehabilitation strategies.

Finally, confounding factors such as treatment variations during hospitalization and socioeconomic influences were not fully controlled and may have impacted outcomes. Collecting detailed clinical and demographic data and applying advanced statistical methods, including propensity score matching and sensitivity analyses, will help minimize bias in future work. Across these limitations, standardized longitudinal phenotyping beyond 24 months and harmonized analytic methods will be important for comparability across cohorts.

## Conclusion

These findings highlight the importance of long-term neurological monitoring, domain-specific cognitive evaluation, and the need for mechanistic studies incorporating neuroimaging to further elucidate the neural substrates of long COVID. Identifying distinct clinical phenotypes based on symptom trajectories may guide future risk stratification, rehabilitation, and intervention strategies aimed at mitigating the enduring impact of Neuro-PASC.

## Data Availability

The original contributions presented in the study are included in the article/Supplementary material, further inquiries can be directed to the corresponding author.
