# Supplementary material for: Longitudinal trajectories of neurologic symptoms and cognitive associations at 36-months post-hospitalization for COVID-19
Source: Front Neurol. 2026 Aug 5;17:1882710. doi: 10.3389/fneur.2026.1882710 (PMC13486164; doi:10.3389/fneur.2026.1882710)
Supplement: Supplementary file 1 [file Table_1.docx]

Supplementary Material

Supplementary Table 1. Baseline characteristics, biomarkers at baseline, anxiety, depression, and cognitive scores during follow-up (N=227)

| Characteristics | N (%) /  median [IQR] | Characteristics | N (%) /  median [IQR] |
| --- | --- | --- | --- |
| Demographics | | GAD, 24M | 3 [0,8] |
| Age | 48 [38,57] | GAD, 36M | 3 [0,6] |
| Sex | | Depression | |
| Female | 108 (47.6) | PHQ, 3M | 5.5 [2,11.5] |
| Male | 119 (52.4) | PHQ, 12M | 5 [2,11] |
| Race | | PHQ, 24M | 5 [2,10] |
| White | 115 (50.7) | PHQ, 36M | 5.5 [2,9] |
| Black or African American | 51 (22.5) | Cognition subscale | |
| American Indian/Alaska Native | 1 (0.4) | TRTA, 3M | 93 [56,104] |
| Asian | 7 (3.1) | TRTA, 12M | 96.5 [82,105] |
| Hawaiian or Pacific Islander | 1 (0.4) | TRTA, 24M | 98 [84,107] |
| Other | 51 (22.5) | TRTA, 36M | 97.5 [82,109] |
| Unknown or not reported | 1 (0.4) | TRTB, 3M | 91 [75,104] |
| Hispanic | | TRTB, 12M | 99 [83,107] |
| No | 90 (39.7) | TRTB, 24M | 96 [83,107] |
| Yes | 137 (60.4) | TRTB, 36M | 99 [86,108.5] |
| Obesity | | SCWT, 3M | 89 [72,106] |
| No | 87 (38.3) | SCWT, 12M | 94.5 [74.5,106.5] |
| Yes | 140 (61.7) | SCWT, 24M | 98 [87, 108] |
| Diabetes type II | | SCWT, 36M | 101 [88, 107] |
| No | 158 (69.6) | IRT, 3M | 97 [82,107] |
| Yes | 69 (30.4) | IRT, 12M | 101 [79,113] |
| Hyperlipidemia | | IRT, 24M | 101 [88, 108] |
| No | 201 (88.6) | IRT, 36M | 102 [90, 113] |
| Yes | 26 (11.5) | DRT, 3M | 94 [77,107] |
| SARS-CoV-2 severity | | DRT, 12M | 102 [84,111] |
| Moderate | 118 (55.1) | DRT, 24M | 102 [88,111] |
| Severe | 85 (39.7) | DRT, 36M | 103 [90,114] |
| Critical | 11 (5.1) | DSST, 3M | 90 [70,105] |
| Anxiety | | DSST, 12M | 91 [68,105] |
| GAD, 3M | 4 [0.5,7] | DSST, 24M | 98 [82,107] |
| GAD, 12M | 3 [0,7] | DSST, 36M | 101 [83,109] |

PHQ, Patient Health Questionnaire-9, GAD, Generalized Anxiety Disorder-7, TRTA, Trail Making test A, TRTB, Trail Making test B, SCWT, Stroop color and word test, IRT, Immediate Recognition test, DRT, Delayed Recognition test, DSST, Digit-Symbol Substitution test

Supplementary Table 2. Results of multivariable generalized estimating equations models for each self-reported neurologic symptom

| Variable | Coordination impairment | Dizziness | Fatigue | Headache | Memory concentration | Sleep issues |
| --- | --- | --- | --- | --- | --- | --- |
| time | 1.112  (1.022-1.209)* | 1.006  (0.932-1.085) | 1.064  (0.965-1.174) | 1.066  (0.982-1.159) | 1.075  (1.000-1.155)* | 0.944  (0.858-1.039) |
| GAD | 0.965  (0.857-1.086) | 0.909  (0.813-1.018) | 0.988  (0.847-1.151) | 1.116  (0.998-1.249) | 1.064  (0.945-1.198) | 1.052  (0.916-1.208) |
| PHQ | 1.171  (1.044-1.314)** | 1.209  (1.086-1.346)*** | 1.168  (1.012-1.347)* | 1.048  (0.942-1.166) | 1.226  (1.100-1.367)*** | 1.115  (0.984-1.264) |
| BrainCheck | 1.006  (0.987-1.026) | 0.991  (0.976-1.006) | 1.001  (0.984-1.019) | 1.003  (0.985-1.022) | 0.996  (0.982-1.010) | 0.992  (0.975-1.010) |
| Age | 1.035  (1.011-1.058)** | 1.017  (0.998-1.037) | 1.030  (1.008-1.053)** | 0.974  (0.954-0.995)* | 1.014  (0.994-1.034) | 1.002  (0.978-1.027) |
| Female | 1.869  (1.030-3.393)* | 1.671  (1.006-2.774)* | 1.270  (0.691-2.336) | 1.868  (1.075-3.246)* | 2.170  (1.264-3.726)** | 1.433  (0.746-2.752) |
| time: GAD | 1.001  (0.996-1.007) | 1.005  (0.999-1.010) | 1.005  (0.996-1.013) | 0.995  (0.990-1.000)* | 1.001  (0.996-1.007) | 1.001  (0.994-1.008) |
| time: PHQ | 0.999  (0.993-1.004) | 0.997  (0.992-1.002) | 1.003  (0.995-1.011) | 1.003  (0.998-1.009) | 0.996  (0.991-1.001) | 1.004  (0.997-1.010) |
| time: BrainCheck | 0.999  (0.999-1.000) | 1.000  (1.000-1.001) | 0.999  (0.998-1.000) | 1.000  (0.999-1.001) | 1.000  (0.999-1.000) | 1.001  (1.000-1.002) |

*, *P*-value < 0.5, **, *P*-value <0.01, ***, *P*-value <0.001. PHQ, Patient Health Questionnaire-9, GAD, Generalized Anxiety Disorder-7.

Supplementary Table 3. Clustering metrics for K-means clustering with longitudinal self-reported neurologic symptom data

| Symptom | Number of clustering, k | Within sum of squares | Between sum of squares | Silhouette score | Calinski-Harabasz Index |
| --- | --- | --- | --- | --- | --- |
| Coordination impairment | 2 | 48.31 | 33.31 | 0.54 | 11.03 |
|  | 3 | 38.49 | 43.13 | 0.56 | 8.40 |
|  | 4 | 30.20 | 51.42 | 0.56 | 7.94 |
| Dizziness | 2 | 63.65 | 34.11 | 0.41 | 8.58 |
|  | 3 | 49.09 | 48.67 | 0.43 | 7.44 |
|  | 4 | 37.64 | 60.12 | 0.47 | 7.45 |
| Fatigue | 2 | 72.26 | 36.70 | 0.32 | 8.13 |
|  | 3 | 59.94 | 49.02 | 0.31 | 6.13 |
|  | 4 | 49.49 | 59.47 | 0.33 | 5.61 |
| Headache | 2 | 54.43 | 30.50 | 0.50 | 8.97 |
|  | 3 | 42.29 | 42.64 | 0.52 | 7.56 |
|  | 4 | 32.17 | 52.76 | 0.55 | 7.65 |
| Memory concentration | 2 | 72.73 | 38.43 | 0.32 | 8.45 |
|  | 3 | 60.61 | 50.54 | 0.27 | 6.25 |
|  | 4 | 51.59 | 59.57 | 0.29 | 5.39 |
| Sleep issues | 2 | 70.37 | 40.06 | 0.33 | 9.11 |
|  | 3 | 57.01 | 53.42 | 0.34 | 7.03 |
|  | 4 | 46.74 | 63.69 | 0.33 | 6.36 |

Supplementary Table 4. Baseline characteristics during hospitalization by neurologic symptom trajectories (Full Results)

| **Characteristics** | **Consistently Low**  **(Group 1)** | **Worsening**  **(Group 2)** | **Improving**  **(Group 3)** | ***p*-value** |
| --- | --- | --- | --- | --- |
| **Coordination impairment** | | | | |
| Age, mean (SD) | 46.4 (12.6) | 51.3 (12.9) | 52.3 (14.8) | 0.011 |
| Male, n (%) | 97 (56.4) | 13 (36.1) | 9 (47.4) | 0.079 |
| Race, n (%) |  |  |  | 0.154 |
| American Indian/Alaska Native | 1 (0.6) | 0 (0) | 0 (0) |  |
| Asian | 4 (2.3) | 2 (5.6) | 1 (5.3) |  |
| Black or African American | 34 (19.8) | 10 (27.8) | 7 (36.8) |  |
| Hawaiian or Pacific Islander | 1 (0.6) | 0 (0) | 0 (0) |  |
| Other | 38 (22.1) | 9 (25) | 4 (21.1) |  |
| Unknown or not reported | 0 (0) | 0 (0) | 1 (5.3) |  |
| White | 94 (54.7) | 15 (41.7) | 6 (31.6) |  |
| Hispanic, n (%) | 108 (62.8) | 22 (61.1) | 7 (36.8) | 0.095 |
| Obesity, n (%) | 105 (61) | 21 (58.3) | 14 (73.7) | 0.522 |
| Diabetes II, n (%) | 45 (26.2) | 18 (50) | 6 (31.6) | 0.024 |
| Hyperlipidemia, n (%) | 16 (9.3) | 6 (16.7) | 4 (21.1) | 0.143 |
| Hypertension, n (%) | 53 (30.8) | 22 (61.1) | 11 (57.9) | 0.002 |
| Asthma, n (%) | 11 (6.8) | 6 (17.6) | 6 (33.3) | 0.006 |
| ARDS, n (%) | 41 (25.3) | 7 (20.6) | 1 (5.6) | 0.151 |
| Severity, n (%) |  |  |  | 0.074 |
| Critical | 6 (3.7) | 4 (11.8) | 1 (5.6) |  |
| Moderate | 85 (52.5) | 20 (58.8) | 13 (72.2) |  |
| Severe | 71 (43.8) | 10 (29.4) | 4 (22.2) |  |
| **Dizziness** | | | | |
| Age, mean (SD) | 46.3 (13.1) | 48.5 (13.3) | 52.7 (11) | 0.010 |
| Male, n (%) | 86 (58.1) | 23 (48.9) | 10 (31.2) | 0.019 |
| Race, n (%) |  |  |  | 0.584 |
| American Indian/Alaska Native | 1 (0.7) | 0 (0) | 0 (0) |  |
| Asian | 5 (3.4) | 2 (4.3) | 0 (0) |  |
| Black or African American | 32 (21.6) | 10 (21.3) | 9 (28.1) |  |
| Hawaiian or Pacific Islander | 1 (0.7) | 0 (0) | 0 (0) |  |
| Other | 36 (24.3) | 7 (14.9) | 8 (25) |  |
| Unknown or not reported | 0 (0) | 0 (0) | 1 (3.1) |  |
| White | 73 (49.3) | 28 (59.6) | 14 (43.8) |  |
| Hispanic, n (%) | 85 (57.4) | 32 (68.1) | 20 (62.5) | 0.436 |
| Obesity, n (%) | 84 (56.8) | 35 (74.5) | 21 (65.6) | 0.093 |
| Diabetes II, n (%) | 43 (29.1) | 16 (34) | 10 (31.2) | 0.788 |
| Hyperlipidemia, n (%) | 12 (8.1) | 8 (17) | 6 (18.8) | 0.078 |
| Hypertension, n (%) | 46 (31.1) | 22 (46.8) | 18 (56.2) | 0.015 |
| Asthma, n (%) | 11 (7.9) | 7 (15.9) | 5 (16.7) | 0.152 |
| ARDS, n (%) | 33 (23.6) | 11 (25) | 5 (16.7) | 0.690 |
| Severity, n (%) |  |  |  | 0.028 |
| Critical | 7 (5) | 1 (2.3) | 3 (10) |  |
| Moderate | 68 (48.6) | 32 (72.7) | 18 (60) |  |
| Severe | 65 (46.4) | 11 (25) | 9 (30) |  |
| **Headache** | | | | |
| Age, mean (SD) | 48.7 (13) | 45.7 (13.9) | 43.4 (10.8) | 0.031 |
| Male, n (%) | 93 (55.4) | 16 (50) | 10 (37) | 0.213 |
| Race, n (%) |  |  |  | 0.944 |
| American Indian/Alaska Native | 1 (0.6) | 0 (0) | 0 (0) |  |
| Asian | 6 (3.6) | 0 (0) | 1 (3.7) |  |
| Black or African American | 39 (23.2) | 5 (15.6) | 7 (25.9) |  |
| Hawaiian or Pacific Islander | 1 (0.6) | 0 (0) | 0 (0) |  |
| Other | 38 (22.6) | 8 (25) | 5 (18.5) |  |
| Unknown or not reported | 1 (0.6) | 0 (0) | 0 (0) |  |
| White | 82 (48.8) | 19 (59.4) | 14 (51.9) |  |
| Hispanic, n (%) | 95 (56.5) | 25 (78.1) | 17 (63) | 0.078 |
| Obesity, n (%) | 100 (59.5) | 20 (62.5) | 20 (74.1) | 0.375 |
| Diabetes II, n (%) | 50 (29.8) | 11 (34.4) | 8 (29.6) | 0.865 |
| Hyperlipidemia, n (%) | 21 (12.5) | 2 (6.2) | 3 (11.1) | 0.689 |
| Hypertension, n (%) | 62 (36.9) | 13 (40.6) | 11 (40.7) | 0.855 |
| Asthma, n (%) | 15 (9.6) | 2 (6.7) | 6 (22.2) | 0.162 |
| ARDS, n (%) | 38 (24.2) | 6 (20) | 5 (18.5) | 0.809 |
| Severity, n (%) |  |  |  | 0.621 |
| Critical | 7 (4.5) | 2 (6.7) | 2 (7.4) |  |
| Moderate | 86 (54.8) | 19 (63.3) | 13 (48.1) |  |
| Severe | 64 (40.8) | 9 (30) | 12 (44.4) |  |

ARDS, Acute Respiratory Distress Syndrome.
